# Demographic and clinical predictors of progression and mortality in connective tissue disease-associated interstitial lung disease: a retrospective cohort study

**DOI:** 10.1186/s12890-019-0943-2

**Published:** 2019-10-31

**Authors:** Chrystal Chan, Christopher J. Ryerson, James V. Dunne, Pearce G. Wilcox

**Affiliations:** 1 Department of Medicine, University of British Columbia, Vancouver, Canada; 2 Centre for Heart Lung Innovation, University of British Columbia, Vancouver, Canada

**Keywords:** Interstitial lung disease, Connective tissue disease, Prognosis, Survival

## Abstract

**Background:**

Connective tissue disease-associated interstitial lung disease (CTD-ILD) is associated with reduced quality of life and poor prognosis. Prior studies have not identified a consistent combination of variables that accurately predict prognosis in CTD-ILD. The objective of this study was to identify baseline demographic and clinical characteristics that are associated with progression and mortality in CTD-ILD.

**Methods:**

Patients were retrospectively identified from an adult CTD-ILD clinic. The predictive significance of baseline variables on serial forced vital capacity (FVC), diffusion capacity (DLCO), and six-minute walk distance (6MWD) was assessed using linear mixed effects models, and Cox regression analysis was performed to assess impact on mortality.

**Results:**

359 patients were included in the study. Median follow-up time was 4.0 (IQR 1.5–7.6) years. On both unadjusted and multivariable analysis, male sex and South Asian ethnicity were associated with decline in FVC. Male sex, positive smoking history, and diagnosis of systemic sclerosis (SSc) vs. other CTD were associated with decline in DLCO. Male sex and usual interstitial pneumonia (UIP) pattern predicted decline in 6MWD. There were 85 (23.7%) deaths. Male sex, older age, First Nations ethnicity, and a diagnosis of systemic sclerosis vs. rheumatoid arthritis were predictors of mortality on unadjusted and multivariable analysis.

**Conclusion:**

Male sex, older age, smoking, South Asian or First Nations ethnicity, and UIP pattern predicted decline in lung function and/or mortality in CTD-ILD. Further longitudinal studies may add to current clinical prediction models for prognostication in CTD-ILD.

## Background

Interstitial lung disease (ILD) is frequently seen in association with rheumatic diseases. The prevalence of ILD varies with disease subtype; ILD is reported in up to 90% of patients with systemic sclerosis (SSc), whereas it is less prevalent in rheumatoid arthritis (RA, 4–68%), mixed connective tissue disease (MCTD, 20–85%), and the inflammatory myopathies polymyositis and dermatomyositis (PM/DM, 15–70%), although reported numbers vary [1–5]. These disorders are collectively termed connective tissue disease-associated interstitial lung disease (CTD-ILD). The majority of CTD-ILD patients display a pattern of nonspecific interstitial pneumonia (NSIP) on high-resolution computed tomography (HRCT) and histopathology, with the exception of patients with RA-ILD, who have an approximately equal proportion of patients with NSIP and usual interstitial pneumonia (UIP).

Although there are differences between CTD subtypes, the presence of ILD is associated with reduced quality of life and worse prognosis [6]. Pulmonary fibrosis is the leading cause of death in patients with SSc and inflammatory myositis, and patients with RA-UIP have a five-year survival rate of 37% [7–9]. Male sex, older age, baseline severity of lung function impairment, and decline in physiologic parameters over time are associated with disease progression and mortality in studies of individual CTD-ILD subtypes [4, 6, 10–13]; however, these studies have not identified a consistent combination of variables that accurately predict prognosis in CTD-ILD. Identification of such variables could have a substantial impact on patient care by identifying patients who might warrant more aggressive therapy or earlier referral for lung transplantation assessment.

The primary objective of this study was to use a longitudinal cohort of patients with CTD-ILD to determine the effect of baseline demographic and clinical variables on change in lung function and mortality. Particularly, we were interested in the prognostic significance of easily attainable demographic variables such as ethnicity and smoking status, which have not been consistently shown to affect prognosis in CTD-ILD in prior literature.

## Methods

### Study population

Patients were retrospectively identified from a specialized adult CTD-ILD clinic between July 2011 and June 2017. The clinic utilizes a multidisciplinary team consisting of a respirologist, rheumatologist, and specialized nurse, with a particular focus on SSc-ILD. Patients were diagnosed based on standard American College of Rheumatology/European League against Rheumatism criteria [14–16] and had ILD on HRCT scan as read by an experienced chest radiologist. Patients provided written informed consent for inclusion in a prospective database (Providence Health Care Research Ethics Board H17–01082).

### Data collection

Demographic variables were obtained from questionnaires at the time of initial ILD clinic visit and extraction from medical chart review. Annual income in Canadian dollars (CAD) was approximated by postal code using data from the 2011 Census and National Household Survey in a database by Environics Analytics [17]. Clinical data including CTD diagnosis, radiographic pattern, and autoantibody status were ascertained from chart and database review. Vital status was determined at the time of data extraction by medical chart review. Patients who underwent lung transplantation were censored at the time of transplantation. Patients underwent pulmonary function tests (PFTs) according to established criteria for measurement of spirometry, lung volumes, and diffusion capacity [18, 19]. Patients completed 6-min walk tests (6MWTs) following established procedures, including use of a forehead saturation probe when appropriate [20]. PFTs and 6MWTs were typically performed at 6-month intervals and HRCT annually, however this was left to the discretion of the treating physician.

### Statistical analysis

Data are presented as mean ± standard deviation, median (interquartile range), or number (%). Continuous data were tested for normality using the Kolmogorov-Smirnov test. Measures of disease progression included %-predicted forced vital capacity (FVC), %-predicted diffusing capacity (DLCO), six-minute walk distance (6MWD), and mortality. Candidate predictor variables were determined a priori, including age at presentation, sex, ethnicity, smoking history (past or current), estimated annual income, CTD subtype (SSc, RA, MCTD, or other CTDs), baseline lung function, and radiographic pattern.

Linear mixed effects models were used to identify predictors of change in FVC, DLCO, and 6MWD over time, with analyses restricted to patients with at least three data points for the outcome of interest. Unadjusted analysis was performed to estimate the rate of change in outcomes for each covariate, and the difference in the rate of change between covariates was assessed. Multivariable analysis was then used to estimate the rate of change adjusted for the other covariates.

The Kaplan-Meier method was used to visualize the survival probability by covariates, and the log-rank test used to compare survival curves. Unadjusted and multivariable Cox regression analysis was then performed to assess the impact of the predictor variables on mortality, with results presented as hazard ratios (HR). All analyses were performed using SAS 9.4 software. *p* < 0.05 was considered statistically significant.

## Results

A total of 359 patients were identified from the database. Patient characteristics are summarized in Table 1. There were 207 patients with SSc-ILD, 45 with RA-ILD, 26 with MCTD-ILD, and 81 with other CTD-ILD. The other CTD-ILD group included patients with polymyositis (*n* = 8), dermatomyositis (*n* = 7), systemic lupus erythematosus (*n* = 13), primary Sjogren’s syndrome (n = 8), interstitial pneumonia with autoimmune features (*n* = 14), and undifferentiated connective tissue disease (n = 13).

**Table 1:** Baseline patient characteristics

Variable	n	All (*n* = 359)	SSc (*n* = 207)	RA (*n* = 45)	MCTD (*n* = 26)	Other (*n* = 81)^a^
Age at first visit, y	357	56 ± 13	55 ± 13	62 ± 14	49 ± 12	58 ± 12
Male, n (%)	359	81 (23)	40 (19)	10 (22)	3 (12)	28 (35)
Ethnicity, n (%)	357					
Caucasian		223 (63)	143 (69)	22 (50)	12 (46)	46 (57)
Asian		62 (17)	28 (14)	5 (11)	9 (35)	20 (25)
South Asian		33 (9)	17 (8)	8 (18)	2 (8)	6 (7)
First nations		24 (7)	12 (6)	4 (9)	3 (12)	5 (6)
Other		15 (4)	6 (3)	5 (11)	0 (0)	4 (5)
Positive smoking history, n (%)	359	180 (50)	94 (45)	24 (53)	12 (46)	50 (62)
Estimated annual income, $	359	80,135 ± 38,403	81,138 ± 41,542	81,850 ± 40,798	74,125 ± 32,098	78,549 ± 29,996
Baseline lung function						
FVC, %-predicted	350	77 ± 20	79 ± 21	75 ± 23	79 ± 16	70 ± 18
DLCO, %-predicted	336	56 ± 19	57 ± 19	56 ± 19	56 ± 18	54 ± 17^b^
6MWD, metres	279	387 ± 123	395 ± 122	312 ± 116	430 ± 117	374 ± 116
Radiographic pattern, n (%)	359					
NSIP Pattern		242 (67)	173 (84)	11 (24)	14 (54)	44 (54)
UIP Pattern		46 (13)	17 (8)	17 (38)	2 (8)	10 (12)
Other/Not specified		71 (20)	17 (8)	17 (38)	10 (39)	27 (33)
Mortality, n (%)	357	85 (24)	66 (32)	6 (13)	3 (12)	10 (12)
Median follow up time, y (IQR)	359	4 (2, 8)	5 (2, 8)	3 (1, 5)	3 (1, 8)	3 (1, 5)

Values are reported as mean ± SD unless otherwise stated. *SSc* systemic sclerosis, *RA* rheumatoid arthritis, *MCTD* mixed connective tissue disease, *FVC* forced vital capacity, *DLCO* diffusing capacity of lungs for carbon monoxide; *6MWD* six-minute walk distance, *UIP* usual interstitial pneumonia, *NSIP* non-specific interstitial pneumonia, *IQR* interquartile range

^a^Polymyositis (*n* = 8), dermatomyositis (*n* = 7), systemic lupus erythematosus (*n* = 13), primary Sjögren’s syndrome (*n* = 8), interstitial pneumonia with autoimmune features (*n* = 14), undifferentiated connective tissue disease (*n* = 31)

^b^Data not normally distributed; median and interquartile range are 52.0 (45.0, 60.0)

### Factors associated with FVC decline

There were 289 patients with at least three FVC measures available for analysis (Table 2). FVC declined at a mean rate of 1.4%-predicted per year (95% confidence interval [CI] 0.9 to 1.8%). On unadjusted analysis, male sex, South Asian ethnicity, and higher income were associated with accelerated decline in FVC. Men had a mean FVC decline of 2.7% per year (95% CI 1.8 to 3.6%) compared to 1.0% per year in women (95% CI 0.5 to 1.4%), and South Asian patients declined 1.7% per year faster than patients of non-South Asian ethnicity (95% CI 0.1 to 3.3%). On multivariable analysis, male sex and South Asian ethnicity remained independent predictors of accelerated decline in FVC.

**Table 2:** Difference in rate of change per year of FVC, DLCO, and 6MWD per covariate in patients with CTD-ILD

Variable	FVC				DLCO				6MWD			
	Unadjusted analysis		Multivariable analysis		Unadjusted analysis		Multivariable analysis		Unadjusted analysis		Multivariable analysis	
	Difference in rate of change (95% CI)	*p*	Difference in rate of change (95% CI)	*p*	Difference in rate of change (95% CI)	*p*	Difference in rate of change (95% CI)	*p*	Difference in rate of change (95% CI)	*p*	Difference in rate of change (95% CI)	*p*
Overall change per year	− 1.4 (− 1.8, − 0.9)	< 0.001	–	–	− 1.8 (− 2.2, − 1.4)	< 0.001	–	–	−9.9 (− 16.0, − 3.8)	0.002	–	–
Age per 10y increase^a^	− 0.2 (− 0.6, 0.1)	0.16	− 0.1 (− 0.5, 0.3)	0.62	− 0.4 (− 0.7, 0.0)	0.03	− 0.3 (− 0.7, 0.0)	0.08	−4.5 (− 9.5, 0.5)	0.08	− 3.1 (−8.6, 2.3)	0.26
Male vs. female	− 1.7 (− 2.7, − 0.7)	< 0.001	− 1.9 (− 3.0, − 0.8)	< 0.001	− 1.1 (− 2.1, − 0.1)	0.03	− 1.3 (− 2.4, − 0.3)	0.02	− 26.6 (− 41.0, − 12.2)	< 0.001	− 28.6 (− 44.5, − 12.6)	< 0.001
Ethnicity												
Caucasian vs. non-Caucasian	−0.4 (− 1.3, 0.5)	0.40	0.1 (− 1.0, 1.2)	0.86	− 0.5 (− 1.4, 0.5)	0.31	0.4 (− 0.7, 1.5)	0.46	−5.0 (− 21.1, 11.2)	0.55	− 8.8 (− 26.1, 8.4)	0.32
EA vs. non-EA	0.5 (− 0.6, 1.7)	0.37	0.5 (− 0.8, 1.8)	0.44	0.5 (− 0.7, 1.7)	0.40	0.1 (− 1.2, 1.3)	0.92	0.5 (− 18.9, 20.0)	0.96	− 1.7 (− 21.6, 18.3)	0.87
SA vs. non-SA	− 1.7 (− 3.3, − 0.1)	0.04	−2.3 (− 4.0, 0.5)	0.010	−0.7 (− 2.4, 1.0)	0.43	−1.9 (− 3.7, − 0.1)	0.04	0.8 (− 24.1, 25.7)	0.95	− 11.6 (− 38.2, 14.9)	0.39
FN vs. non-FN	1.5 (− 0.4, 3.3)	0.12	0.8 (− 1.2, 2.8)	0.44	0.5 (− 1.3, 2.2)	0.60	0.2 (− 1.7, 2.1)	0.86	0.9 (− 34.3, 36.2)	0.96	5.6 (− 32.0, 43.2)	0.77
Pos. vs. neg. Smoking	− 0.2 (− 1.1, 0.6)	0.61	−0.2 (− 1.1, 0.8)	0.75	−1.0 (− 1.8, − 0.2)	0.02	−1.1 (− 2.0, − 0.2)	0.02	−0.4 (− 12.6, 11.8)	0.95	3.7 (− 10.2, 17.6)	0.60
Income per 10 K increase	−0.2 (− 0.4, 0.0)	0.02	− 0.1 (− 0.3, 0.1)	0.20	−0.1 (− 0.3, 0.0)	0.14	−0.1 (− 0.2, 0.1)	0.55	−1.7 (− 4.3, 1.0)	0.21	−0.2 (− 3.1, 2.7)	0.90
CTD subtype												
SSc vs. RA	0.3 (−1.1, 1.7)	0.67	−0.1 (− 2.0, 1.7)	0.90	0.2 (−1.2, 1.6)	0.76	−0.1 (− 2.0, 1.8)	0.93	18.2 (− 12.2, 48.6)	0.24	18.6 (−22.4, 59.5)	0.37
SSc vs. MCTD	0.0 (−1.7, 1.7)	0.99	0.1 (−1.8, 1.9)	0.96	−0.6 (−2.2, 0.9)	0.43	−0.8 (−2.5, 1.0)	0.40	−17.7 (− 43.0, 7.6)	0.17	− 18.7 (− 45.4, 7.9)	0.17
SSc vs. other CTD	− 1.0 (− 2.1, 0.1)	0.08	−1.8 (− 3.1, − 0.5)	0.006	−1.5 (− 2.6, − 0.4)	0.007	−2.0 (− 3.2, − 0.7)	0.002	−9.0 (− 31.0, 13.1)	0.43	−23.3 (− 47.9, 1.2)	0.06
Baseline value^a^ (per 10%-predicted / 100 m decrease)	0.0 (− 0.2, 0.2)	0.93	0.1 (− 0.2, 0.3)	0.69	0.2 (0.0, 0.4)	0.09	0.2 (− 0.1, 0.4)	0.14	2.5 (−3.2, 8.3)	0.39	3.5 (−2.8, 9.8)	0.28
UIP vs. NSIP	−0.9 (−2.4, 0.6)	0.23	− 0.7 (− 2.3, 0.9)	0.39	0.1 (− 1.4, 1.6)	0.88	0.6 (− 1.1, 2.2)	0.49	− 28.9 (− 50.7, − 7.0)	0.010	− 24.2 (− 46.9, − 1.5)	0.04

*FVC* forced vital capacity; *DLCO* diffusing capacity of lungs for carbon monoxide, *6MWD* six-minute walk distance, *EA* East Asian, *SA* South Asian, *FN* First Nations, *SSc* systemic sclerosis, *RA* rheumatoid arthritis, *MCTD* mixed connective tissue disease, *CTD* connective tissue disease, *UIP* usual interstitial pneumonia, *NSIP* non-specific interstitial pneumonia

Multivariable analyses performed using linear mixed effects models were adjusted for sex, age, ethnicity, estimated income, smoking history, CTD subtype, radiographic pattern, anti-nuclear antibody status, baseline FVC/DLCO/6MWD

^a^Modelled as continuous variables but reported in increments of 10 years (age), 10%-predicted (FVC, DLCO), 100 m (6MWD) for illustrative purposes

### Factors associated with DLCO decline

There were 262 patients with at least three DLCO measures available for analysis (Table 2). DLCO declined at a mean rate of 1.8%-predicted per year (95% CI 1.4 to 2.2%). On unadjusted analysis, male sex, older age, positive smoking history were significant predictors of decline in DLCO. When stratified by CTD subtype (SSc, RA, MCTD, and other CTDs), diagnosis of SSc compared to other CTDs was a significant predictor of decline in DLCO. Men had a DLCO decline of 2.6% per year (95% CI 1.8 to 3.5%) compared to 1.6% per year in women (95% CI 1.1 to 2.0%), and smokers 2.3% per year (95% CI 1.7 to 2.9%) compared to 1.3% per year in non-smokers (95% CI 0.8 to 1.9%). DLCO declined by 0.4% per year more for every 10 years’ increase in age at first presentation (95% CI 0.0 to 0.7%). DLCO of SSc-ILD patients declined at a rate of 2.1% per year (95% CI 1.6 to 2.5%), RA-ILD at 2.3% per year (95% CI 1.0 to 3.6%), MCTD-ILD at 1.4% per year (95% CI 0.1 to 2.9%), and other CTD-ILD at 0.6% per year (95% CI 0.4 to 1.5%). On multivariable analysis, male sex, positive smoking history, and diagnosis of SSc vs. other CTDs remained independent predictors of decline in DLCO.

### Factors associated with 6MWD decline

There were 181 patients with at least three 6MWT measures available for analysis (Table 2). 6MWD decreased at a mean rate of 9.9 m per year (95% CI 3.8 m to 16.0 m). On unadjusted analysis, male sex and UIP pattern predicted accelerated decline in 6MWD. 6MWD declined at a rate of 30.9 m per year in men (95% CI 18.1 m to 43.7 m) compared to 4.3 m per year in women (95% CI − 2.3 m to 11.0 m), and 34.9 m per year for patients with UIP pattern (95% CI 14.0 m to 55.7 m) compared to 6.0 m per year for patients with NSIP pattern (95% CI 0.6 m to 12.7 m). On multivariable analysis, both male sex and UIP pattern remained independent predictors of accelerated decline in 6MWD.

### Mortality

There were 85 (23.8%) deaths among the 357 patients with follow-up data after the initial consult (Table 3). The mean age at death was 63.9 ± 14.5 years. Among deceased patients, 20 (23.5%) were male, 59 (69.4%) were Caucasian, 42 (49.4%) had a history of smoking, and 66 (77.6%) had a diagnosis of SSc. On HRCT, 51 (60.0%) had a NSIP pattern and 18 (21.1%) had a UIP pattern.

**Table 3:** Predictors of mortality in CTD-ILD

Variable	Unadjusted analysis		Multivariable analysis	
	Hazard ratio	*p*	Hazard ratio	*p*
	(95% CI)		(95% CI)	
Age per 10y increase	1.03 (1.01, 1.05)	< 0.001	1.0 (1.0, 1.1)	0.002
Male vs. female	1.8 (1.1, 3.0)	0.03	2.5 (1.2, 4.9)	0.010
Ethnicity				
Caucasian vs. non-Caucasian	2.1 (1.1, 3.9)	0.03	1.4 (0.6, 3.4)	0.51
EA vs. non-EA	0.7 (0.3, 1.7)	0.48	0.5 (0.1, 1.4)	0.17
SA vs. non-SA	0.9 (0.3, 2.4)	0.81	1.1 (0.3, 3.9)	0.94
FN vs. non-FN	3.2 (1.3, 7.5)	0.009	4.7 (1.3, 16.4)	0.02
Pos. vs. neg. Smoking	1.2 (0.8, 1.9)	0.32	1.1 (0.6, 1.9)	0.85
Income per 10 K increase	1.0 (0.9, 1.1)	0.53	1.0 (0.9, 1.1)	0.71
CTD subtype				
SSc vs. RA	2.6 (1.1, 6.2)	0.04	10.4 (1.6, 67.1)	0.014
SSc vs. MCTD	2.2 (0.7, 7.1)	0.17	1.1 (0.3, 4.3)	0.88
SSc vs. other CTD	1.9 (1.0, 3.7)	0.07	4.1 (1.2, 13.3)	0.02
Baseline FVC (per 10% decrease)	1.1 (1.0, 1.2)	0.06	1.2 (1.0, 1.4)	0.10
Baseline DLCO (per 10% decrease)	1.3 (1.1, 1.5)	< 0.001	1.2 (1.0, 1.5)	0.08
Baseline 6MWD (per 100 m decrease)	1.4 (1.1, 1.7)	0.005	1.1 (0.8, 1.4)	0.74
UIP vs. NSIP	2.3 (1.4, 4.0)	0.002	0.9 (0.4, 2.1)	0.80

*CTD* connective tissue disease, *ILD* interstitial lung disease, *EA* East Asian, *SA* South Asian, *FN* First Nations, *SSc* systemic sclerosis, *RA* rheumatoid arthritis, *MCTD* mixed connective tissue disease, *UIP* usual interstitial pneumonia, *NSIP* non-specific interstitial pneumonia

**Fig. 1:**
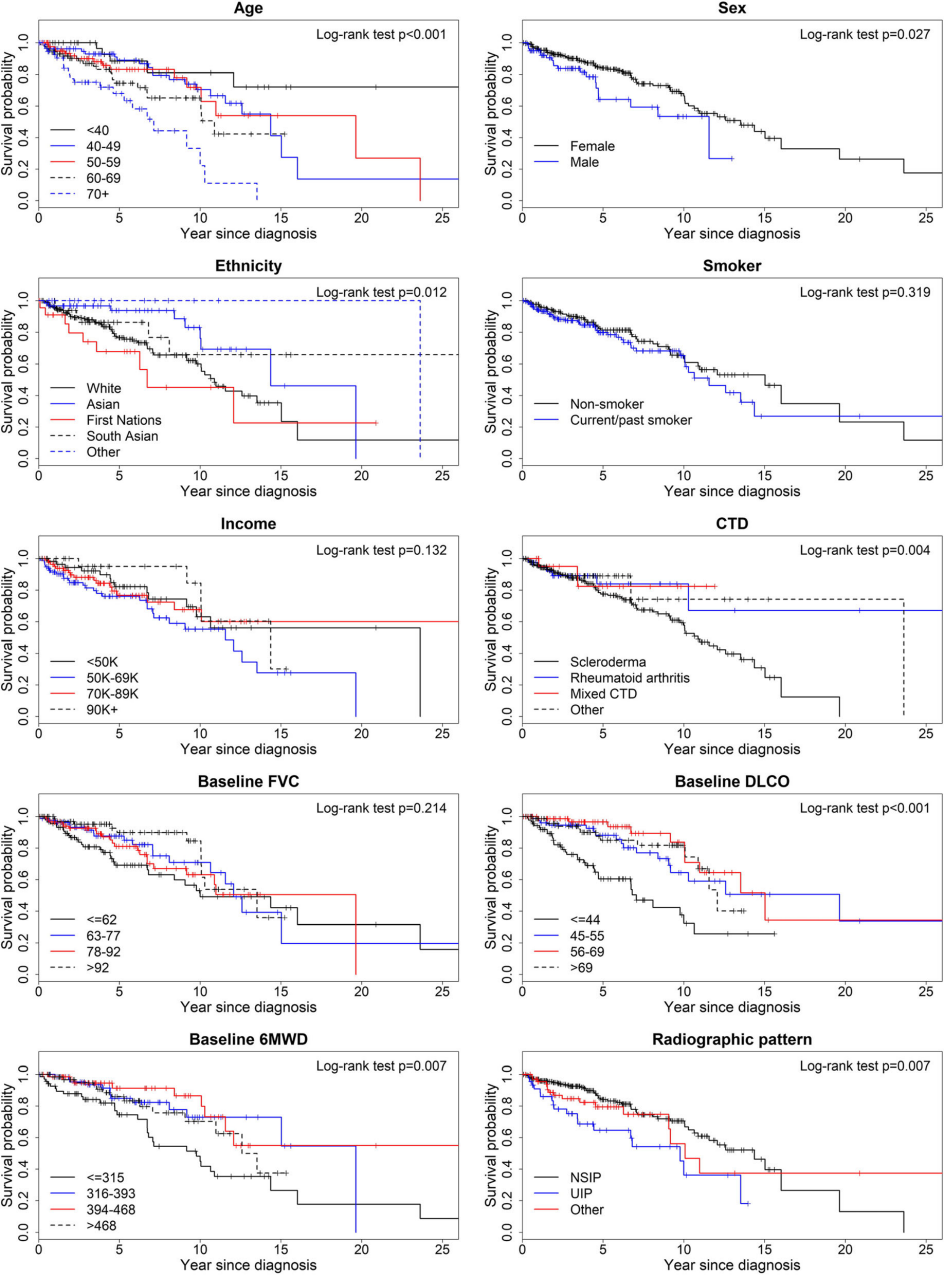
Kaplan-Meier curves depicting effect of baseline predictors variables on survival

The Kaplan-Meier survival curves were significantly different on log-rank test when comparing sex, age at presentation, ethnicity, CTD subtype, radiographic pattern, baseline DLCO, and baseline 6MWD (Fig. 1). Unadjusted Cox regression analysis identified increased mortality in males compared to females (HR 1.8, 95% CI 1.1 to 3.0), SSc compared to RA (HR 2.6, 95% CI 1.1 to 6.2), and UIP compared to NSIP pattern (HR 2.3, 95% CI 1.4 to 4.0). Older age at presentation was also predictive of mortality, with HR 1.03 (95% CI 1.01 to 1.05) for every 10 years’ increase in age. Caucasian ethnicity (HR 2.1, 95% CI 1.1 to 3.9) and First Nations ethnicity (HR 3.2, 95% CI 1.3 to 7.5) were additional predictors of mortality compared to non-Caucasian and non-First Nations ethnicity respectively. Lower baseline DLCO and lower baseline 6MWD were predictors of mortality, with HR 1.3 (95% CI 1.1 to 1.5) for every 10%-predicted decrease in baseline DLCO and HR 1.4 (95% CI 1.1 to 1.7) for every 100 m decrease in baseline 6MWD. When multivariable analysis using Cox proportional-hazard model was performed, male sex, older age, and First Nations ethnicity remained independent risk factors for mortality. As well, patients with SSc-ILD had higher mortality compared with patients with RA-ILD.

## Discussion

Our study represents a comprehensive analysis of patients with CTD-ILD evaluated at our tertiary care centre. Patient characteristics were similar to previously reported cohorts of CTD-ILD, apart from a somewhat higher proportion of SSc-ILD and lower proportion of RA-ILD patients, likely related to differences in referral patterns [21–23]. Five-year survival in our cohort was 80% with median survival 12.6 years, which is similar to or better than other cohorts [22, 24].

Our results support previous studies that showed that male sex and UIP pattern are independent predictors of disease progression and mortality in CTD-ILD. Our finding that male sex predicts decline in FVC, DLCO, and 6MWD has not been consistently reported in other studies that evaluated predictors of lung function decline [10, 11, 13, 25–27]. Additionally, we found that UIP pattern was associated with accelerated 6MWD decline on both unadjusted and multivariable analysis. UIP pattern is a well documented predictor of progression and mortality in RA-ILD [11, 12, 28], has been associated with ILD progression in PM/DM-ILD in one study [13], and has potential prognostic ability in SSc-ILD [10].

When stratified by CTD (SSc vs. RA vs. MCTD vs. other CTDs), patients with SSc-ILD had accelerated DLCO decline compared to other CTD-ILD, and increased mortality compared to RA-ILD. One small study performed in South Korea involving 93 CTD-ILD patients found no difference in mortality between CTD subtypes [24], and multiple previous studies have shown improved survival in SSc-ILD compared to that in other CTD-ILD [21, 22, 29], with particularly poor survival in in RA-ILD [5, 30]. The reason for the discordance in our cohort is unclear but is likely related to methodologic differences between studies or relatively small numbers of non-SSc patients in our cohort, particularly RA-ILD. Additionally, we did not assess for pulmonary hypertension in our study, which is seen in association with SSc and is a known predictor of mortality and lung function decline [8, 10]. However, the discrepancy in our cohort seems to be driven by improved survival of the non-SSc-ILD patients, as survival of SSc-ILD patients in our cohort was comparable to or even higher than that of prior studies [21, 29].

Interestingly, patients of South Asian ethnicity had accelerated decline in FVC compared to patients of non-South Asian ethnicity. This has not been previously demonstrated, and results may be biased by the small numbers in this subgroup, however this may represent a combination of genetic, ecological, and exposure factors. This did not translate to an increase in mortality, possibly due to inadequate power to detect this difference. In one study of 70 SSc patients in the United Kingdom, the prevalence of ILD was twice as high in South Asian patients compared to Caucasian patients, however they did not assess lung function decline in established CTD-ILD [31]. An ILD registry in India that included 151 CTD-ILD patients found higher numbers of RA-ILD compared to SSc-ILD and a relatively greater proportion of UIP in their cohort [32], factors which have been associated with poorer prognosis, however these variables were controlled for in our analysis. Overall, the impact of ethnicity on CTD-ILD is not well studied [33, 34]. Most epidemiologic studies of CTD-ILD have been done in the United States or Europe with predominantly Caucasian, black, and Hispanic patients [22, 25, 32, 35] No previous studies have specifically noted the increased mortality in patients of First Nations ethnicity with CTD-ILD as was seen in our cohort. However, given that we did not ascertain cause of death, this finding must be taken in context with the well-documented disproportionate burden of mortality among First Nations people [36]. One systematic review of patients with CTD found that mortality in patients of First Nations ethnicity is frequently attributable to disease progression and complications, however the proportional attribution of CTD severity and social factors to mortality has not been evaluated [37].

Positive smoking history was predictive of faster decline in DLCO, likely in part driven by patients with concomitant emphysema. Most studies have shown that smoking is not an independent risk factor for mortality or disease progression CTD-ILD [4, 10, 11, 28, 38–40], however smoking is included in a proposed risk prediction model for CTD-ILD that also includes age, DLCO, and pulmonary vessel volume [23]. In our cohort, smoking was not an independent predictor for mortality.

Previous studies have identified additional predictors of disease progression and mortality within CTD subtypes, many of which are outside the scope of our study [4, 10, 12, 41]. A clinical prediction model based on such variables could identify high-risk patients who may warrant closer surveillance, more aggressive therapy, or earlier referral for lung transplant. A risk prediction model incorporating sex, age, and DLCO, and another model incorporating FVC, DLCO, and forced expiratory volume in one second (FEV1) have been shown to predict mortality in SSc-ILD, and a modified version of the GAP index has been shown to predict mortality in CTD-ILD with reasonable accuracy [39, 42, 43]. In addition to these variables, our results support consideration of ethnicity and CTD subtype as additional prognostic factors, although further research in this area is needed.

Our study is limited by the analysis of patients from a single tertiary care centre, which may not be generalizable to community centres or other academic institutions. There was a high proportion of patients with SSc-ILD. Our study was also limited by sample size for some comparisons and may be underpowered to identify more subtle predictors of prognosis. Furthermore, it may be possible that some of the observed associations are false-positive findings, as we did not correct for multiple comparisons. The retrospective nature of our study resulted in missing or inadequate data, including cause of death. We were unable to assess certain known predictive factors for ILD progression such as disease duration, longitudinal disease activity, pulmonary hypertension, or HRCT severity. Lastly, survivorship bias may influence longitudinal models. Despite these limitations, this is one of the few studies to predict longitudinal change in PFTs using easily attainable predictor variables in a diverse CTD-ILD population, and is the first to evaluate the effect of certain ethnicities on disease progression.

## Conclusion

Our data support prior studies that show that male sex, older age, a history of smoking, and UIP pattern predict progression and mortality in CTD-ILD. We additionally identified novel risk factors including South Asian and First Nations ethnicity. We hope that these data can be used to inform discussions between patients and clinicians around treatment decisions. Given the substantial morbidity and mortality associated with CTD-ILD, further longitudinal studies may add to current clinical prediction models for prognostication in CTD-ILD.

## Data Availability

The datasets used and analysed during the current study are available from the corresponding author on reasonable request.

## Abbreviations

6MWD: six-minute walk distance
6MWT: six-minute walk test
CAD: Canadian dollars
CTD: Connective tissue disease
DLCO: Diffusion capacity of the lungs for carbon monoxide
FVC: Forced vital capacity
HR: Hazard ratio
HRCT: High-resolution computed tomography
ILD: Interstitial lung disease
MCTD: Mixed connective tissue disease
NSIP: Nonspecific interstitial pneumonia
PFT: Pulmonary function tests
PM/DM: Polymyositis/dermatomyositis
RA: Rheumatoid arthritis
SSc: Systemic sclerosis
UIP: Usual interstitial pneumonia
